# Dynamic Input Conductances Shape Neuronal Spiking^1^,^2^

**DOI:** 10.1523/ENEURO.0031-14.2015

**Published:** 2015-03-25

**Authors:** Guillaume Drion, Alessio Franci, Julie Dethier, Rodolphe Sepulchre

**Affiliations:** 1 Systems and Modeling, Department of Electrical Engineering and Computer Science, University of Liège, Liège, B-4000, Belgium; 2 Laboratory of Pharmacology and GIGA Neurosciences, University of Liège, Liège, B-4000, Belgium; 3 Volen Center and Biology Department, Brandeis University, Waltham, Massachussetts 02454; 4 Department of Engineering, University of Cambridge, Cambridge, CB2 1PZ, United Kingdom; 5 Department of Mechanical and Aerospace Engineering, Princeton University, Princeton, New Jersey 08544

**Keywords:** compensation, firing pattern, ion channels, neuromodulation

## Abstract

Assessing the role of biophysical parameter variations in neuronal activity is critical to the understanding of modulation, robustness, and homeostasis of neuronal signalling. The paper proposes that this question can be addressed through the analysis of dynamic input conductances. Those voltage-dependent curves aggregate the concomitant activity of all ion channels in distinct timescales. They are shown to shape the current−voltage dynamical relationships that determine neuronal spiking. We propose an experimental protocol to measure dynamic input conductances in neurons. In addition, we provide a computational method to extract dynamic input conductances from arbitrary conductance-based models and to analyze their sensitivity to arbitrary parameters. We illustrate the relevance of the proposed approach for modulation, compensation, and robustness studies in a published neuron model based on data of the stomatogastric ganglion of the crab *Cancer borealis*.

## Abstract (precis)

Reliable neuron activity is ensured by a tight regulation of the ion channels that resides in the neuron’s membrane. Understanding the causal mechanisms that relate this regulation to physiological and pathological neuronal activity is a necessary step for developing efficient therapies for neurological diseases associated with abnormal nervous system activity.

## Significance Statement

Reliable neuron activity is ensured by a tight regulation of the ion channels that resides in the neuron’s membrane. Understanding the causal mechanisms that relate this regulation to physiological and pathological neuronal activity is a necessary step for developing efficient therapies for neurological diseases associated with abnormal nervous system activity. Our paper provides a novel methodological framework to quantify the sensitivity of neuronal activity to changes in ion channel densities. This framework, which is general and can be applied to any neuron type, has the potential to improve our understanding of the regulation of brain functions and to help in the design of new pharmacological treatments.

## Introduction

Neuron membrane potential and spiking result from the dynamical interplay of many different ion channels, whose gating kinetics span a broad spectrum of voltage ranges and timescales (Hille, 1984). From this complexity arises the specificity of each neuronal type as well as an abundance of modulation possibilities. These underlie the richness of signaling in the nervous system (Harris-Warrick and Marder, 1991; Bargmann, 2012; Marder, 2012; Nusbaum and Blitz, 2012; Nadim and Bucher, 2014). At the same time, neuronal activity is highly robust and adaptable to changing environments (Swensen and Bean, 2005; Beverly et al., 2011). Moreover, it is increasingly clear that the same neuronal activity can be produced in spite of large variability in biophysical parameters such as ion channel densities or half-activation potentials (Goldman et al., 2001; Prinz et al., 2004; Schultz et al., 2006; Taylor et al., 2009; Marder 2011; Amendola et al., 2012; Marder et al., 2014). This robustness underlies the amazing stability and adaptability of the nervous system. Understanding the ionic mechanisms that can simultaneously support modulation and robustness of neuron activity has been, and remains, an important focus of contemporary neurophysiology.

The mechanisms underlying ion channel interplay in generating different firing patterns are of particular interest for understanding neuromodulation and compensation. Deepening our understanding of those mechanisms through methodological tools could lead to the development of more effective treatments for neurological disorders associated with abnormal brain activity (Goaillard and Dufour, 2014). Although great advances in this area have been achieved by influential experimental and computational studies over the last decades (Goldman et al., 2001; Grashow et al., 2009; Marder, 2011; O’Leary et al., 2013; O’Leary et al., 2014; Prinz et al., 2004; Schultz et al., 2006; Swensen and Bean, 2005; Taylor et al., 2009), some important questions still remain unsettled. Specifically, our intuition concerning firing pattern sensitivity to changes in ion channel densities or the ability of some channels to compensate for the loss of others remains empirical and qualitative rather than systematic and quantitative, and expensive and numerous experiments are usually required to answer such challenging inquiries (Achard and De Schutter, 2006; Prinz, 2007; Prinz, 2010; Doloc-Mihu and Calabrese, 2014).

The present paper proposes an innovative avenue of attack to investigate the mechanisms of ion channel interplay in shaping neuronal spiking. It shows that the dynamical gating of the different ion channels is linked to neuron firing activity through rigorous quantities: the dynamic input conductances. Dynamic input conductances are voltage-dependent curves that aggregate the role of all ion channels in the generation of each distinct temporal event that characterizes firing activity (three in a bursting neuron: fast for spike upstroke, slow for spike downstroke and interspike period, and ultraslow for spike adaptation and interburst period). These curves shape the current−voltage dynamical relationships that determine neuronal spiking.

We show that the sensitivity of neuronal activity with respect to a particular biophysical parameter such as ion channel density correlates with the ability of this parameter to shape one or several dynamic input conductances in specific voltage ranges. From a modulation viewpoint, these data are directly relevant to interpreting and predicting the conductance targets of neuromodulators from how they affect the neuronal activity, and vice versa. From a robustness viewpoint, we show that the colocalization of the sensitivity ranges of different ion channels into specific timescales is necessary and sufficient to allow for large parameter variability nonetheless producing fixed neuronal activity due to simple compensation mechanism, regardless of other ion channel particulars.

We provide a computational method to extract dynamic input conductances from an arbitrary conductance-based model and perform a sensitivity analysis of neuronal activity to arbitrary parameter variations on those dynamic input conductances. Throughout this paper, we illustrate its predictive value in a specific conductance-based model that has served many previous experimental and computational studies (Turrigiano et al., 1995; Liu et al., 1998; Goldman et al., 2001). These data show the generality of the proposed approach and suggest it has relevance to assist experimental studies of neuronal modulation and robustness. The proposed computational tool is appealing in its mathematical simplicity, which facilitates the interpretation of the results and allows for a systematic sensitivity analysis even in high-dimensional models. In addition, we provide a voltage-clamp protocol to directly measure dynamic input conductances in real neurons. Simulated experiments show that both methods give very similar results.

## Materials and Methods

### STG neuron model

Membrane currents are described in Goldman et al. (2001). The kinetics and voltage dependence of the conductances contributing to the currents are based on measurements of crustacean STG neurons (Turrigiano et al., 1995). All parameters are similar to the ones given in Goldman et al. (2001), except for the calcium reversal potential, which is fixed here to +120 mV. The model is composed of six different ionic currents: *I*_Na_, *I*_Ca,T_, *I*_Ca,S_, *I*_K,A_, *I*_K,d_, and *I*_K,Ca_.

### Computation of the static and dynamic input conductances

Static and dynamic input conductances of a neuron model composed of variables *X_i_* are computed as follows $g_{f}=\frac{\partial I_{f}}{\partial V}=\underset{i}{\sum }w_{fs}^{X_{i}}\left(\frac{\partial \overset{˙}{V}}{\partial X_{i}}\frac{\partial X_{i,\infty }}{\partial V}\right),$ $g_{s}=\frac{\partial I_{s}}{\partial V}=\underset{i}{\sum }\left(w_{su}^{X_{i}}-w_{fs}^{X_{i}}\right)\left(\frac{\partial \overset{˙}{V}}{\partial X_{i}}\frac{\partial X_{i,\infty }}{\partial V}\right),$ $g_{u}=\frac{\partial I_{u}}{\partial V}=\underset{i}{\sum }\left(1-w_{su}^{X_{i}}\right)\left(\frac{\partial \overset{˙}{V}}{\partial X_{i}}\frac{\partial X_{i,\infty }}{\partial V}\right),$ $g=g_{f}+g_{s}+g_{u}=\underset{i}{\sum }\left(\frac{\partial \overset{˙}{V}}{\partial X_{i}}\frac{\partial X_{i,\infty }}{\partial V}\right),$where $w_{fs}^{X_{i}}$ and $w_{su}^{X_{i}}$ are voltage-dependent weighting factors that determine the contribution of the variable *X_i_* in the fast (*f*), slow (*s*) and ultraslow (*u*) timescales. They are defined as logarithmic distances between the variable time constant $\tau_{X_{i}}$ and the fast, slow, and ultraslow time constants. Note that static and dynamic input conductances are voltage-dependent, which is not explicitly written in the equations for clarity purposes. We do not include the passive properties of the membrane, i.e. the term $\frac{\partial \dot{V}}{\partial V}$, in the computation of the static and dynamic input conductances, as those variations are almost instantaneous compared to those induced by ion channel kinetics.

The fast, slow, and ultraslow time constants are extracted as follows: the fast time constant τ*_f_* (*V_m_*) corresponds to the activation time constant of the fastest depolarizing current (*I*_Na_ in the STG neuron model, $\tau_{f}\left(V_{m}\right)=\tau_{m_{Na}}\left(V_{m}\right)$). The slow time constant τ*_s_*(V*_m_*) corresponds to the activation time constant of the fastest repolarizing current (*I*_K,d_ in the STG neuron model, $\tau_{s}\left(V_{m}\right)=\tau_{m_{K,d}}\left(V_{m}\right)$), the kinetics of this current giving the upper frequency limit of fast spiking. The ultraslow time constant τ*_u_*(V*_m_*) corresponds to the time constant of the slowest variable (which is the inactivation of I_Ca,S_ in the STG neuron model, $\tau_{u}\left(V_{m}\right)=\tau_{h_{Ca,S}}\left(V_{m}\right)$). The two voltage-dependent weighting factors *w_fs_^X_i_^* and *w_su_^X_i_^* are assigned to all other variables *X_i_*, as follows:

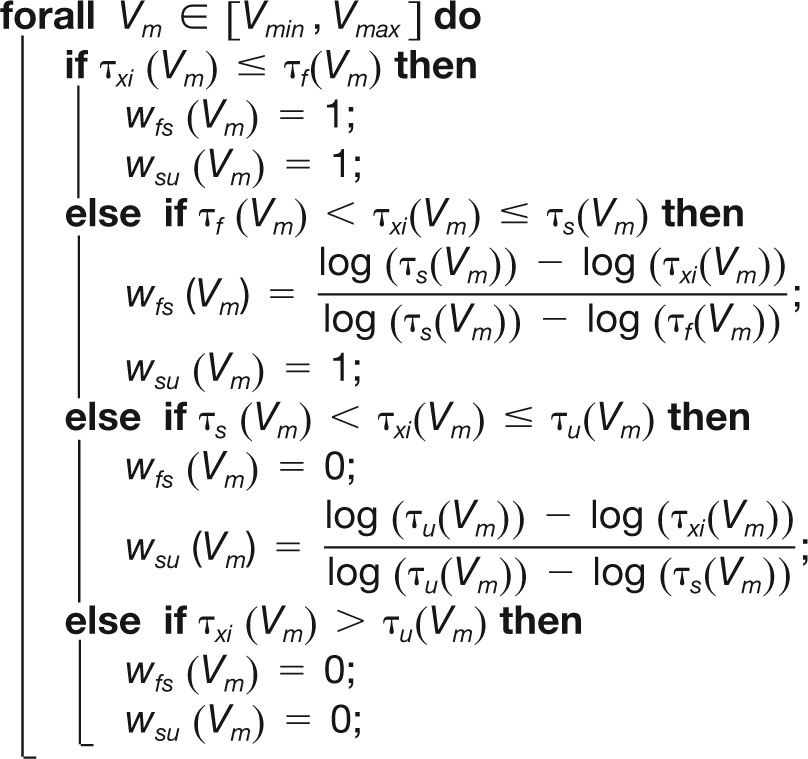



**Fig. 1:**
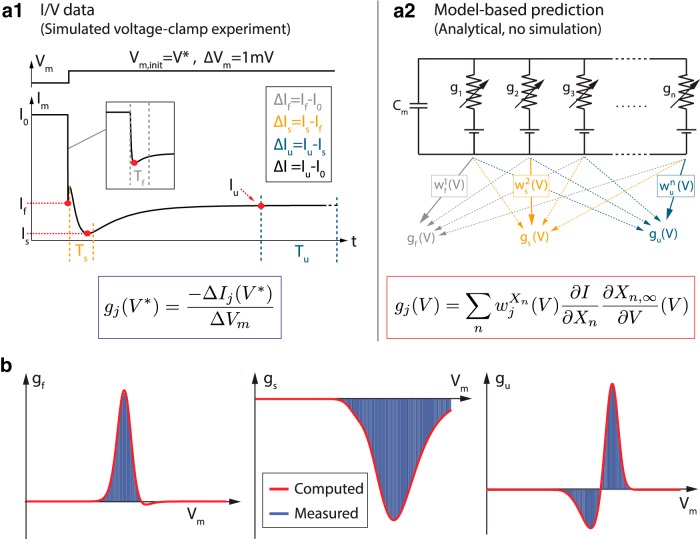
Dynamic input conductances shape neuron dynamic sensitivity. ***a1***, Example of an experimental measurement of dynamic input conductances in voltage-clamp. A step of potential ΔV (top) induces a variation in the transmembrane current ΔI (bottom). The values of the currents playing a role in the different timescales are extracted as shown on the figure. ***a2***, Sketch of the mathematical derivation of the dynamic input conductances from an arbitrary conductance-based model. The dynamic input conductances are computed by aggregating the role of the different ionic conductances in each timescale. ***b***, Dynamic input conductances of a STG neuron model for a particular set of parameters either measured in a simulated voltage-clamp experiment (blue bars) or computed following the described mathematical procedure (red lines).

Values of STG model maximal conductances used to generate the dynamic input conductances shown in Figs. 1 and 3 are *ḡ*_Na_ = 700 mS/cm^2^, *ḡ_Ca_*_,_*_T_* = 2 mS/cm^2^, *ḡ_Ca_*_,_*_S_* = 4 mS/cm^2^, *ḡ_A_* = 50 mS/cm^2^, *ḡ_K_*_,_*_d_* = 70 mS/cm^2^, and *ḡ_K_*_,_*_Ca_* = 40 mS/cm^2^. Note that intracellular calcium plays a dynamical role in the ultraslow timescale only (intracellular calcium concentration is an ultraslow integrator of calcium entry, which itself depends on the slow activation of the voltage-gated calcium channels).

### Sensitivity analysis of the dynamic input conductances

The sensitivity curves are computed by taking the derivative between the input conductances and each ion channel maximal conductance *ḡ_i_*. For an arbitrary ionic current of the form $I_{i}\;=\;{\bar{g}}_{i}m_{i}^{p}h_{i}^{q}\;(V_{m}\;-\;V_{i})$, it gives

$$\frac{\partial g_{f}}{\partial {\overset{¯}{g}}_{i}}=w_{fs}^{m_{i}}pm_{i}^{p-1}h_{i}^{q}\left(V_{m}-V_{i}\right)\frac{\partial m_{i,\infty }}{\partial V}+w_{fs}^{h_{i}}m_{i}^{p}qh_{i}^{q-1}\left(V_{m}-V_{i}\right)\frac{\partial h_{i,\infty }}{\partial V},$$

$$\frac{\partial g_{s}}{\partial {\overset{¯}{g}}_{i}}=(w_{su}^{m_{i}}-w_{fs}^{m_{i}})pm_{i}^{p-1}h_{i}^{q}\left(V_{m}-V_{i}\right)\frac{\partial m_{i,\infty }}{\partial V}+(w_{su}^{h_{i}}-w_{fs}^{h_{i}})m_{i}^{p}qh_{i}^{q-1}\left(V_{m}-V_{i}\right)\frac{\partial h_{i,\infty }}{\partial V},$$

$$\frac{\partial g_{u}}{\partial {\overset{¯}{g}}_{i}}=(1-w_{su}^{m_{i}})pm_{i}^{p-1}h_{i}^{q}\left(V_{m}-V_{i}\right)\frac{\partial m_{i,\infty }}{\partial V}+(1-w_{su}^{h_{i}})m_{i}^{p}qh_{i}^{q-1}\left(V_{m}-V_{i}\right)\frac{\partial h_{i,\infty }}{\partial V}.$$

Again, these sensitivity curves depend on the membrane potential, which is not explicitly written in the equations for clarity purposes.

Once these sensitivity functions are computed, we extract their value at spike threshold (*V_th_*) and up-state (*V_osc_*). To rigorously extract spike threshold, we apply the algorithm described in Franci et al. (2013). This algorithm detects a transcritical bifurcation in arbitrary conductance-based models. This bifurcation point is the point of maximum sensitivity, where the steady-state potential of the model neuron is exactly at spike threshold. We choose to vary the maximal conductance of one of the calcium channels to detect this bifurcation in the STG neuron model. Spike threshold potential is around –50 mV in the model. To extract up-state, we simply compute the *I*/*V* curve and take the more depolarized zero of the curve. This zero corresponds to the unstable steady-state potential around which oscillations occur. Up-state potential is approximately –16 mV in the model STG neuron. Finally, we sketch the localization of the different sensitivity functions on the *V_m_* axis by normalizing them. This permits comparing these localizations one to one regardless of the amplitude differences.

### Construction of the compensation mechanism based on the sensitivity analysis

For illustration purposes, we derive here the compensation mechanism for changes in only one calcium conductance at a time. This procedure is general and can be extended to perturbations of any conductance or other parameter.

1. First, a set of maximal conductances is chosen to generate the reference firing pattern.
2. Dynamic input conductances *g_f_* (*V_m_*), *g_s_ (V_m_)*, *g_u_ (V_m_)* and the *I*/*V* curve I_static_(V_m_) are computed for this set of maximal conductances following the procedure described above.
3. Maximal conductances (or other parameters) that will be involved in the compensation mechanism are chosen. It is important that this set of parameters is sufficient to cover all the timescales that will be affected by a change in the perturbed conductance. This can be determined via the sensitivity analysis described in the previous section. In this manuscript, we use *ḡ_A_*, *ḡ_K_*_,_*_d_*, *ḡ_K_*_,_*_Ca_* and I_app_ in the compensation mechanism. We did not use *ḡ_Na_* because none of the calcium channels significantly affect the fast timescale in their range of variation.
4. The values of the dynamic input conductances (and *I*/*V* curve) that will be maintained must be defined. The number of different values cannot exceed the number of parameters that are involved in the compensation mechanism. We choose to maintain the following values:$g_{s}^{\text{*}}(V_{th})$, $g_{s}^{\text{*}}(V_{osc})$, $g_{u}^{\text{*}}(V_{th})$, and $I_{static}^{\text{*}}(V_{th})$. V_th_ and V_osc_ are computed as described above.
5. Finally, the set of *ḡ_A_*, *ḡ_K_*_,_*_d_*, *ḡ_K_*_,_*_Ca_*, and *I_app_* that maintains $g_{s}^{\text{*}}(V_{th})$,$g_{s}^{\text{*}}(V_{osc})$,$g_{u}^{\text{*}}(V_{th})$, and $I_{static}^{\text{*}}(V_{th})$ unchanged for each variation of a calcium channel maximal conductance ${\bar{g}}_{Ca}^{▄}$ are computed by solving the linear system *Ax = b* with (example given for ${\overset{¯}{g}}_{Ca}^{▄}={\overset{¯}{g}}_{Ca,S}^{▄})$
  $$A=\left[\begin{array}{cccc} 0 & \frac{\partial g_{s}}{\partial {\overset{¯}{g}}_{K,d}}(V_{th}) & \frac{\partial g_{s}}{\partial {\overset{¯}{g}}_{A}}(V_{th}) & \frac{\partial g_{s}}{\partial {\overset{¯}{g}}_{K,Ca}}(V_{th})\\ 0 & \frac{\partial g_{s}}{\partial {\overset{¯}{g}}_{K,d}}(V_{osc}) & \frac{\partial g_{s}}{\partial {\overset{¯}{g}}_{A}}(V_{osc}) & \frac{\partial g_{s}}{\partial {\overset{¯}{g}}_{K,Ca}}(V_{osc})\\ 0 & \frac{\partial g_{u}}{\partial {\overset{¯}{g}}_{K,d}}(V_{th}) & \frac{\partial g_{u}}{\partial {\overset{¯}{g}}_{A}}(V_{th}) & \frac{\partial g_{u}}{\partial {\overset{¯}{g}}_{K,Ca}}(V_{th})\\ 1 & \frac{\partial I_{static}}{\partial {\overset{¯}{g}}_{K,d}}(V_{th}) & \frac{\partial I_{static}}{\partial {\overset{¯}{g}}_{A}}(V_{th}) & \frac{\partial I_{static}}{\partial {\overset{¯}{g}}_{K,Ca}}(V_{th}) \end{array}\right]x=\left[\begin{array}{c} I_{app}\\ {\overset{¯}{g}}_{K,d}\\ {\overset{¯}{g}}_{A}\\ {\overset{¯}{g}}_{K,Ca} \end{array}\right]$$
  $$b=\left[\begin{array}{c} g_{s}^{*}\left(V_{th}\right)-\left({\overset{¯}{g}}_{Na}\frac{\partial g_{s}}{\partial {\overset{¯}{g}}_{Na}}\left(V_{th}\right)+{\overset{¯}{g}}_{Ca,T}\frac{\partial g_{s}}{\partial {\overset{¯}{g}}_{Ca,T}}\left(V_{th}\right)+{\overset{¯}{g}}_{Ca,S}^{▄}\frac{\partial g_{s}}{\partial {\overset{¯}{g}}_{Ca,S}}\left(V_{th}\right)\right)\\ g_{s}^{*}\left(V_{osc}\right)-\left({\overset{¯}{g}}_{Na}\frac{\partial g_{s}}{\partial {\overset{¯}{g}}_{Na}}\left(V_{osc}\right)+{\overset{¯}{g}}_{Ca,T}\frac{\partial g_{s}}{\partial {\overset{¯}{g}}_{Ca,T}}\left(V_{osc}\right)+{\overset{¯}{g}}_{Ca,S}^{▄}\frac{\partial g_{s}}{\partial {\overset{¯}{g}}_{Ca,S}}\left(V_{osc}\right)\right)\\ g_{u}^{*}\left(V_{th}\right)-\left({\overset{¯}{g}}_{Na}\frac{\partial g_{u}}{\partial {\overset{¯}{g}}_{Na}}\left(V_{th}\right)+{\overset{¯}{g}}_{Ca,T}\frac{\partial g_{u}}{\partial {\overset{¯}{g}}_{Ca,T}}\left(V_{th}\right)+{\overset{¯}{g}}_{Ca,S}^{▄}\frac{\partial g_{u}}{\partial {\overset{¯}{g}}_{Ca,S}}\left(V_{th}\right)\right)\\ I_{static}^{*}\left(V_{th}\right)-\left({\overset{¯}{g}}_{Na}\frac{\partial I_{static}}{\partial {\overset{¯}{g}}_{Na}}\left(V_{th}\right)+{\overset{¯}{g}}_{Ca,T}\frac{\partial I_{static}}{\partial {\overset{¯}{g}}_{Ca,T}}\left(V_{th}\right)+{\overset{¯}{g}}_{Ca,S}^{▄}\frac{\partial I_{static}}{\partial {\overset{¯}{g}}_{Ca,S}}\left(V_{th}\right)\right) \end{array}\right]$$

### Voltage-clamp measurement of the static and dynamic input conductances

Static and dynamic input conductances can be measured in a voltage-clamp experiment, as shown in Fig. 1b. Neuron membrane potential is initially held at a specific value V^*^. A small step of membrane potential Δ*V* is applied and the temporal variation of the transmembrane currents is recorded and normalized around its initial value (Δ*V* = 1 mV in our simulated voltage-clamp experiments). Three specific values are extracted from transmembrane current variations: the amplitude of the local minimum that occurs within 2 ms after the onset of the step (*I_f_*), the amplitude of the local minimum that occurs between 10 and 100 ms after the onset of the step (*I_s_*) (if no local minimum is found, the amplitude at 10 ms is taken), and the minimal amplitude of current from 1 s until the end of the stimulation (*I_u_*).The dynamic currents are measured as follows: the fast dynamic current Δ*I_f_* corresponds to the difference between the amplitude of the initial current *I_0_* and *I_f_*, the slow dynamic current ΔI_s_ corresponds to the difference between *I_s_* and *I_f_*, and the ultraslow dynamic current Δ*I_u_* corresponds to the difference between *I_u_* and *I_s_*. The static current Δ*I* is derived as usual (difference between the current at steady-state, *I_u_* in our case, and the initial current *I_0_*).The values of the dynamic input conductances are given by $g_{j}\left(V^{\text{*}}+\frac{\Delta V}{2}\right)=\frac{-\Delta I_{j}}{\Delta V}\;$, where *j = f, s, u*.

The protocol is reproduced using different holding potentials.

## Results

### Dynamic input conductances shape dynamic sensitivity

In static conditions, the membrane potential of a neuronal model is determined from Kirchhoff’s law. This static equation of the form *I*(*V*) = 0 is the mathematical basis for a static analysis of the local sensitivity of the resting potential to a current variation Δ*I* via the well-known formula

$$\Delta V={\left(\frac{\partial I}{\partial V}\right)}^{-1}\Delta I\;$$

This formula indicates that the (static) input conductance $g\left(V\right)=-\frac{\partial I}{\partial V}$ shapes the sensitivity to current variations. This shaping is voltage-dependent and the sensitivity is maximal in voltage ranges where *g*(*V*) vanishes or changes sign. The classical voltage-clamp experiment is an experimental method to determine the voltage-dependence of the static input conductance *g*(*V*).

Owing to its dynamical nature, a dynamic analog of Equation 1 is needed to study the sensitivity of neuronal spiking. Dynamical sensitivity analysis normally requires solving a set of linearized differential equations, the sensitivity equations (Khalil, 2002), which is computationally impractical in a high-dimensional conductance-based model. This complexity can be circumvented by exploiting the property that neuronal activity is made of a temporal sequence of events with different timescales. For example, an action potential, or spike, exhibits two distinct timescales: a fast timescale for the spike upstroke, determined by the fastest gating kinetics (i.e., sodium activation), and a slow timescale for the membrane repolarization, determined by the potassium-rectifier activation. Likewise, slow spiking, or bursting, has three timescales: in addition to the two timescales of the action potential, it exhibits a third ultraslow timescale determined by the slowest gating kinetics of the participating ionic currents.

Each spiking event is shaped by the relationship between membrane potential variations and transmembrane current variations in the corresponding timescale. Following Equation 1, the Δ*I* – Δ*V* relationship can be quantified in each timescale *j* by a voltage-dependent conductance *g_j_*(*V*). This conductance aggregates the role of all ion channels acting in this timescale. A key message of this paper is that these voltage-dependent conductances, that we call dynamic input conductances, provide key information about the dynamic sensitivity of neuronal activity in their corresponding timescale. Together with the static information contained in the classical *I*/*V* curve, they shape the nature of excitability.

The value of the dynamic input conductances can be measured experimentally without any *a priori* knowledge of neuron dynamical properties (Fig. 1*a1*). In a voltage-clamp experiment, any current variation Δ*I* generated by a step of membrane potential Δ*V* can be decomposed into three distinct contributions (Fig. 1*a1*; see Materials and Methods for further details)

$${\left(\Delta I\right)}_{f}+{\left(\Delta I\right)}_{s}+{\left(\Delta I\right)}_{u}=\Delta I,$$

where (Δ*I*)*_f_*, (Δ*I*)*_s_*, and (Δ*I*)*_u_* are the fast, slow, and ultraslow dynamical components, respectively. Each current component obeys the sensitivity relationship (Eq. 1), leading to the decomposition

$$-g_{f}\left(V\right)\Delta V-g_{s}\left(V\right)\Delta V-g_{u}\left(V\right)\Delta V=-g\left(V\right)\Delta V\;$$

and in turn

$$g_{f}\left(V\right)+g_{s}\left(V\right)+g_{u}\left(V\right)=g\left(V\right)\;.\;$$

Each of the three conductances appearing in the left hand sides of Equations 2 and 3 is the quasi-static quantity $-\frac{\partial I}{\partial V}$ in one distinct timescale, that is, assuming that the current variations that are fast in that timescale have reached their quasi-steady state, and that current variations that are slow in that timescale can be neglected. This simplification, which permits us to determine the different points where the currents are measured in the experiment (see Materials and Methods), is justified mathematically by singular perturbation theory (Fenichel, 1977). It should be stressed that this analysis is fully consistent with the classical voltage-clamp experiment of Hodgkin-Huxley (1952a, 1952b) when modeling the action potential as a two-timescale phenomenon, with the sodium activation accounting for the fast current variations and potassium activation and sodium inactivation accounting for the slow current variations. In the Hodgkin-Huxley model, the static conductance *g*(*V*) can be decomposed as *g*(*V*) = *g_f_*(*V*) + *g_s_*(*V*), where *g_f_*(*V*) is determined by considering the sodium activation to be at steady-state and regarding the other gating variables as quasi-constant parameters.

Dynamic input conductances can also be computed for an arbitrarily detailed conductance-based model without any simulation/measurement of its temporal evolution. In a realistic conductance-based model such as those developed today for a number of neurons, the numerous gating variables exhibit a continuum of voltage-dependent timescales. This means that a given physiological gating variable can, in principle, contribute to each of the three representative timescales of the overall activity. For this reason, the dynamic input conductance in each representative timescale is expressed as a (voltage-dependent) linear combination of all ionic conductances (Figs. 1*a2*, 2; see Materials and Methods for further details). The three dynamic input conductance *g_f_*(*V*), *g_s_*(*V*), and *g_u_*(*V*) can then be interpreted as aggregate conductances in each of the three timescales defining neuronal activity. It should be stressed that the few timescales of the dynamic input conductances are a characteristic of the neuronal activity only, not of ion channel kinetics.

**Fig. 2:**
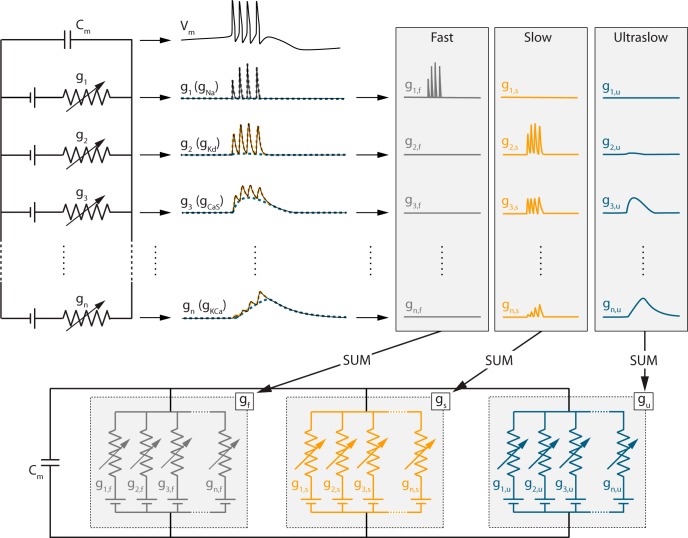
Any ion channel potentially contributes to each of the representative timescales of the membrane potential activity. Top left, Scheme of an arbitrary high-dimensional conductance-based model. Top center, Variations of the membrane potential V_m_ and the different voltage-gated conductances g_i_ over time for a specific set of ion channel densities. Top right, Decomposition of the temporal traces in three different timescales: fast, slow, and ultraslow. Bottom, Reconstruction of the conductance-based model where the contributions of each variable conductance are grouped by timescales, forming the three dynamic input conductances g_f_, g_s_, and g_u_ (see Materials and Methods for details about the rigorous construction).

As an illustration, Fig. 1*b* shows the dynamic input conductances of a STG neuron model (Turrigiano et al., 1995; Liu et al., 1998; Goldman et al., 2001) for a particular set of parameters either measured in a simulated voltage-clamp experiment (blue bars) or computed following the described computational procedure (red lines). It is apparent that both methods provide fully consistent results.

### Voltage-dependent input conductances determine neuron spiking activity

**Fig. 3:**
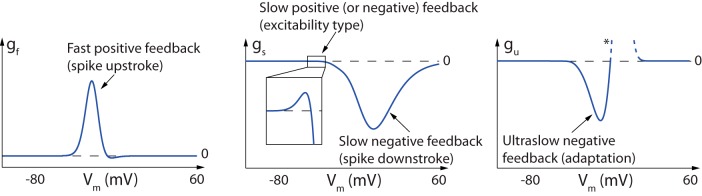
Dynamic input conductances shape neuronal activity. From left to right: fast (g_f_), slow (g_s_), and ultraslow (g_u_) input conductances as a function of the membrane potential V_m_. These curves shape the feedback gain of the neuronal circuit in distinct timescales, thereby determining the dynamical activity.

The three dynamic input conductances computed in Fig. 1 for the particular STG model and reproduced in greater detail in Fig. 3 are typical of a bursting neuron and can be obtained through a great variety of channel combinations. They aggregate the detailed biological information of ionic currents into voltage-dependent curves that shape the dynamical activity of the neuron.

Each curve shapes the voltage-dependent gain of a feedback loop in the associated timescale. In particular, the sign of this feedback gain determines the qualitative role of each feedback loop in a given voltage window. The fast dynamic input conductance is mostly positive. It determines the fast feedback loop of neuronal excitability, which is an excitatory loop corresponding to the fast autocatalytic feedback associated with the action potential regenerative upstroke. The slow dynamic input conductance is mostly negative, with a peak close to the depolarized voltage potential. It determines the slow negative feedback loop of repolarization. However, zooming around the threshold potential shows a small area of positive feedback in the slow timescale. This slow positive feedback is the signature of regenerative excitability, an essential component of the slow excitability that underlies bursting (Franci et al., 2012, 2013, 2014). How ion channels shape the positive area of the slow dynamic input conductance is therefore crucial for the regulation of bursting. The onset of a positive area in the slow dynamic input conductance has a precise mathematical characterization, because it is determined by a transcritical bifurcation that can be easily computed in an arbitrary conductance-based model (Franci et al., 2014). We use this point of maximal sensitivity to determine the excitability threshold of an arbitrary neuronal model. Finally, the ultraslow dynamic input conductance is mostly negative. It shapes the negative feedback loop in the ultraslow timescale and subthreshold voltage potential area where spike adaptation is regulated. The reader will notice that, in this particular model, the ultraslow dynamic input conductance shows an unexpected region of positive feedback at suprathreshold potentials (marked by a * in Fig. 3, right). This positive feedback comes from the interaction between calcium channel inactivations and the dynamical role of intracellular calcium concentration in the ultraslow timescale. However, for the ultraslow timescale (and any slower timescale), the shape of the dynamic input conductance in the suprathreshold region does not influence the neuronal activity because voltage excursions in this region are too fast to recruit the ultraslow feedback.

The simplicity of the dynamic input conductances at a qualitative level explains the ability of simplified dynamical models to reproduce the qualitative properties of neuronal bursting. Two-state models that capture the fast positive feedback and the slow negative feedback each with one-state variable are the essence of excitable models, such as the FitzHugh-Nagumo model or two-variable reductions of the Hodgkin-Huxley model (FitzHugh, 1961; Hindmarsh and Rose, 1984; Rinzel, 1985; Izhikevich, 2007). A three-state dynamical model that captures the qualitative features of the dynamic input conductances shown in Figure 3 has also been recently introduced (Franci et al., 2014): positive feedback in the fast timescale, non-monotone feedback (positive at hyperpolarized potentials and negative at depolarized potentials) in the slow timescale, and negative feedback in the ultraslow timescale. This paper shows why such a dynamical motif organizes the excitability of a bursting neuron and how a restricted number of parameters control the resulting temporal activity. Those parameters only shape the location and amplitudes of the four peaks exhibited in Figure 3.

**Fig. 4:**
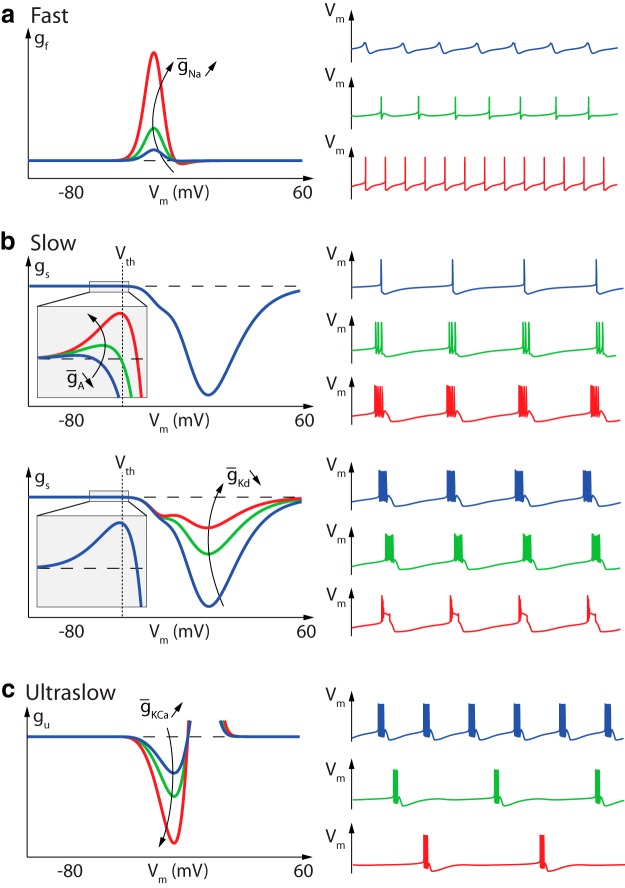
Variations of fast, slow, and ultraslow dynamic input conductances near the threshold potential and peak amplitudes shape spiking activity. ***a***, Fast dynamic input conductance for different values of sodium channel density (left) and associated firing activity in the associated conductance-based model (right). Increased sodium channel density induces an increase in the fast dynamic conductance, which results in an increase in action potential amplitude (up to the saturated value VNa). ***b***, Top, Slow input dynamic conductance for different values of A-type potassium channel density (left) and associated firing activity (right). Changes in A-type potassium channel density affect the value of the slow dynamic conductance at spike threshold, which mainly alters neuron burstiness. Bottom, Slow dynamic input conductance for different values of delayed-rectifier potassium channel density (left) and associated firing activity (right). Changes in delayed-rectifier channel density affects the value of the slow dynamic conductance at up-state, which mainly alters spike repolarization capability. ***c***, Ultraslow dynamic input conductance for different values of calcium-activated potassium channel density (left) and associated firing activity (right). Increased potassium channel density increases the negative peak of the ultraslow dynamic conductance in the subthreshold region, resulting in a decrease in the intraburst frequency.

Figure 4 illustrates how variations in the dynamic input conductances significantly affect the temporal activity. Not surprisingly, Figure 4*a* shows that the fast dynamic input conductance shapes the spike. Increasing the amplitude of the fast positive feedback gain near spike threshold leads to a gradual switch from small oscillatory potentials to tonic spiking. Figure 4*b*, top, illustrates the role of the positive area in the slow dynamic input conductance curve near spike threshold: this area controls the switch from slow restorative to slow regenerative excitability (Franci et al., 2013), that is, the transition from tonic spiking to bursting (Franci et al., 2014). Figure 4*b*, bottom, illustrates the qualitative role of the slow negative feedback at depolarized potentials: reducing the amplitude of the negative peak of the slow dynamic input conductance reduces the negative feedback necessary for spike repolarization, eventually leading to a depolarization block. Finally, burst frequency is mostly controlled by the negative feedback in the ultraslow timescale in the subthreshold region (Fig. 4*c*). In particular, increasing the ultraslow negative feedback concomitantly decreases the length of the bursts and increases the interburst period.

This analysis shows the ability of a qualitative analysis of the dynamic input conductances and their variation to capture nontrivial variations in neuron temporal activity. It is important to note that, although the examples shown in Figure 4 are very simple and straightforward in linking one current to its effect in one timescale, most ion channels contribute to several timescales, and therefore shape several properties of neuronal spiking, as illustrated in the next section. In addition, physiologically relevant neuromodulation often requires the concomitant modulation of dynamics in several timescales. For instance, it is clear from Figure 4 that decreasing burst frequency while maintaining constant duty cycle requires actions on both the slow and ultraslow timescales at least. This supports the fact that most neuromodulators need to target many different ion channel types in order to generate a reliable qualitative change in neuron spiking activity and highlights the relevance of the proposed approach for the identification of such targets.

### Sensitivity analysis of dynamic input conductances predicts how ion channels shape spiking activity

A fundamental property of the voltage-dependent dynamic input conductances analyzed in the preceding section is that they can be quantitatively and algorithmically computed from an arbitrary conductance-based model. As a consequence, they provide a bridge between the quantitative electrophysiology of a given neuron and the control of the few aggregate quantities that shape its dynamical activity. In this section, we illustrate the predictive value of a classical sensitivity analysis of the dynamic input conductances with respect to maximal conductance parameters (i.e., density of a particular channel). Our illustrations are made on the same STG model as in the previous section, but the method is general and elementary from a computational viewpoint, and therefore applicable to any other quantitative neuronal model. All predictions are based on computing sensitivity curves of the type $\frac{\partial g_{f,s,u}}{\partial {\bar{g}}_{x}}(V)$, which evaluates at each membrane potential the derivative of a given dynamic input conductance *g_f,s,u_* with respect to a given maximal conductance parameter *ḡ_x_*. The analysis thus provides one sensitivity curve per channel type and per timescale. The sign and amplitude of this nondimensional quantity in a given voltage range determines how much a given channel can shape the dynamical activity of the neuron in the timescale of the dynamic input conductance *g*(*V*). Below we illustrate the type of predictions that can be made in each of the three timescales of neuronal activity.

**Fig. 5:**
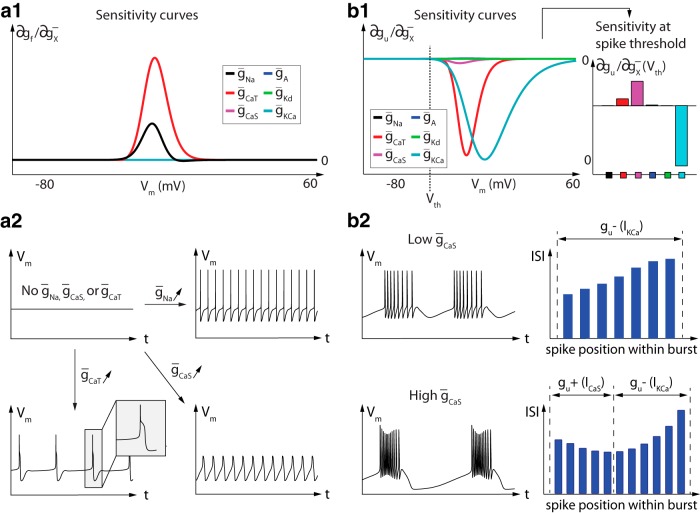
Sensitivity analysis of model STG neuron spiking activity in the fast (left) and ultraslow (right) timescales. The figure illustrates the six sensitivity curves of the STG model in the fast timescale (***a1***) and in the ultraslow timescale (***b1***). The values of the ultraslow sensitivity curves are also plotted at the specific threshold potential, which is a key potential value for excitability properties. ***a2***, Neuronal activity in the absence of sodium and calcium channels (top left), in the presence of sodium channels only (top right), in the presence of T-type calcium channels only (bottom left) and in the presence of slow calcium channels only (bottom right). ***b2***, Neuronal activity (left) and values of the ISIs within each burst (right) of bursters expressing a low (top) and a high (bottom) slow calcium channel density. g_u–_, Ultraslow negative feedback; g_u+_ ultraslow positive feedback.

Figure 5 provides the six sensitivity curves of the model STG neuron in the fast and ultraslow timescales (left and right panels, respectively). Only two ion channel types significantly contribute to the fast conductance: sodium channels and T-type calcium channels (Fig. 5*a1*). The role of sodium channels as the main source of fast positive feedback for spike generation is of course no surprise. Its sensitivity predominates over all other channels at the threshold potential. But the sensitivity analysis in the fast timescale is also instructive regarding the distinctive role of T-type calcium channels with respect to other calcium channels. The sensitivity curves predict that T-type calcium channels participate in the fast excitability properties of the neuron, in contrast to slow calcium channels. The temporal traces in Figure 5*a2* illustrate that in the absence of sodium channels, spikes can be generated with T-type calcium channels only (bottom left panel), due to their contribution in the fast timescale. In contrast, only slow oscillatory potentials can be obtained through slow calcium channels in the absence of sodium and T-type calcium channels. This is because the slow calcium channels make no contribution in the fast timescale.

The six sensitivity curves in the ultraslow timescale show the dominant contribution of three distinct ion channel types: T-type calcium channels, slow calcium channels, and calcium-activated potassium channels (Fig. 5*b1*, left). Those channels mostly cooperate in providing the ultraslow negative feedback necessary for spiking adaptation, through the ultraslow inactivation of calcium channels and the ultraslow activation of potassium channels. This negative feedback is among other things critical for bursting termination. But closer scrutiny of the sensitivities at the threshold potential shows that the slow calcium channels also generate a localized positive feedback, which indicates that their activation also contributes to the ultraslow timescale (Fig. 5*b1*, right). This localized positive feedback in the ultraslow timescale is a source of excitability in the ultraslow timescale, which translates into an increased frequency of spikes during bursts.

Figure 5*b2* confirms this prediction through the comparison of two bursters that differ only in their slow calcium channel density. The burster expressing a low density of slow calcium channels exhibits a monotonically decreasing intraburst frequency [i.e., an increase in the value of the interspike intervals (ISIs); Fig. 5*b2*, top], which indicates that the dynamic input conductance is only negative in the ultraslow timescale. In contrast, the burster expressing a high density of slow calcium channels exhibits a biphasic activity: a period of increasing frequency (i.e., decreasing ISIs) at the beginning of the burst, a consequence of the localized positive feedback brought by the calcium channels in the ultraslow timescale, followed by a period of decreasing frequency (Fig. 5*b2*, bottom). This bursting type is generally referred to as parabolic bursting (Rinzel and Lee, 1987). Its signature in the sensitivity curves is a sufficiently large source of positive feedback in the ultraslow timescale around spike threshold. This signature is quantified by the proposed sensitivity analysis.

**Fig. 6:**
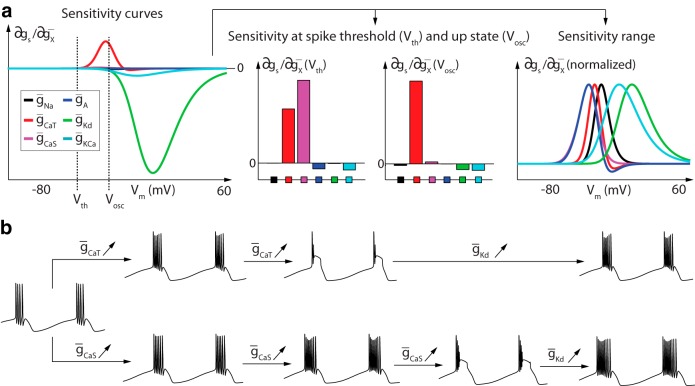
Sensitivity analysis of model STG neuron spiking activity in the slow timescale. The figure shows sensitivity curves that extract the effect of conductance variations on the dynamic input conductances (top) and example of predictions that can be made from these sensitivity curves (bottom). ***a***, Sensitivity of the slow dynamic input conductance for each V_m_ (left), at spike threshold (V_th_), and at up-state (V_osc_) (center) as well as their localization in V_m_ (right). ***b***, Model membrane potential variations over time for different value of *ḡ_Ca,T_*, *ḡ_Ca,S_*, and *ḡ_K,d_*. An increase in both slow and T-type calcium channel densities increases neuron burstiness due to their positive effect at spike threshold. However, an increase in T-type calcium channel density quickly results in depolarization block, due to their positive effect in the up state.

Figure 6*a* shows the six sensitivity curves of the model STG neuron in the slow timescale. It is readily observed that the negative feedback brought by delayed-rectifier potassium channels (green curve) completely dominates the role of any other channel at depolarized potential (Fig. 6*a*, left). This ensures robustness of the action potential downstroke, a key property for robust spiking.

Further scrutiny of the sensitivity values around spike threshold and up-state (Fig. 6*a*, center) allows for finer predictions. Around spike threshold, where small changes in the balance between positive and negative feedback has a strong effect on neuronal spiking (see above), the positive contribution of calcium channels is many-fold higher than the negative contribution of potassium channels. This explains why a tiny calcium current (many-fold weaker than sodium and potassium currents) is sufficient to control excitability in the slow timescale. The simulations in Figure 6*b* confirm that slight changes in calcium conductance control burstiness.

The sensitivity plot at high potential (Fig. 6*a*, center, right) predicts another important difference between T-type calcium channels, whose high sensitivity persists at high potential, and slow calcium channels, whose sensitivity is concentrated near threshold potential. The localized sensitivity of slow calcium channels allows them to control burstiness without affecting spike termination. In contrast, increasing the density of T-type channels concomitantly increases the positive feedback at high potential, conflicting with the negative feedback of delayed-rectifier potassium channels necessary for spike downstroke. Figure 6*b*, center, confirms this prediction of sensitivity analysis: increasing the density of T-type calcium channels quickly leads to depolarization block, which indicates that slow positive feedback has overcome the slow negative feedback at high potential, leading to bistability between the down and up states. Obtaining the same phenomenon with slow calcium channels requires a much larger variation of channel density because of their low sensitivity at high potential.

Not surprisingly, the depolarization block can be eliminated by simultaneously increasing the density of delayed-rectifier potassium channels, which restores the negative feedback at up-state without affecting the balance at spike threshold (Fig. 6*b*, right). A larger conductance for the delayed-rectifier potassium thus increases spiking robustness, protecting the neuron from depolarization block while permitting a broader modulation of intraburst frequency via variations in calcium channel density.

The illustrations in this section stress that a particular ion channel can contribute in more than one timescale and that its effect in different timescales can be studied through different sensitivity curves. For the STG model, slow calcium channels provide an example of current that has no contribution in the fast timescale but contributes a source of excitability concomitantly in the slow timescale (increasing burstiness) and in the ultraslow timescale (parabolic bursting).

Our analysis also illustrates the importance of sensitivity range as well as sensitivity amplitude. Different ion channel types can affect sensitivity in very different ranges, mainly due to different half-activation and inactivation potentials and different ion resting potentials. For the STG model, the sensitivity range is an important source of differentiation between the role of T-type and slow calcium channels in the slow timescale.

### Sensitivity analysis predicts possible compensation mechanisms for robustness and homeostasis

Sensitivity curves accurately predict how varying the density of a given ion channel type affects the dynamic input conductances and how this voltage-dependent shaping affects neuronal activity. At the same time, they predict how other channels can compensate for a parameter variation in order to minimize the change in dynamic input conductances. This insight is important for the quantification of robustness and homeostatic mechanisms that govern neuronal spiking.

We tested this prediction in the STG model by studying how variations in the two calcium maximal conductances (*ḡ_Ca,T_* and *ḡ_Ca,S_*) could be compensated for by variations in the potassium channel conductances (*ḡ_A_*, *ḡ_K,d_*, and *ḡ_K,Ca_*). Our elementary compensation mechanism determines the necessary parameter variations to maintain three distinct values of the dynamic input conductances: the slow input conductance at spike threshold and up-state, and the ultraslow input conductance at spike threshold. In addition to the conductance parameter variations, the external applied current was adjusted to maintain a constant value of the static *I*/*V* curve at spike threshold. Physiological compensation for the static current would possibly require additional ionic currents not present in the STG model. It should also be noted that the absence of static current compensation does not significantly affect the robustness of the dynamic compensation mechanism (simulations not shown).

**Fig. 7:**
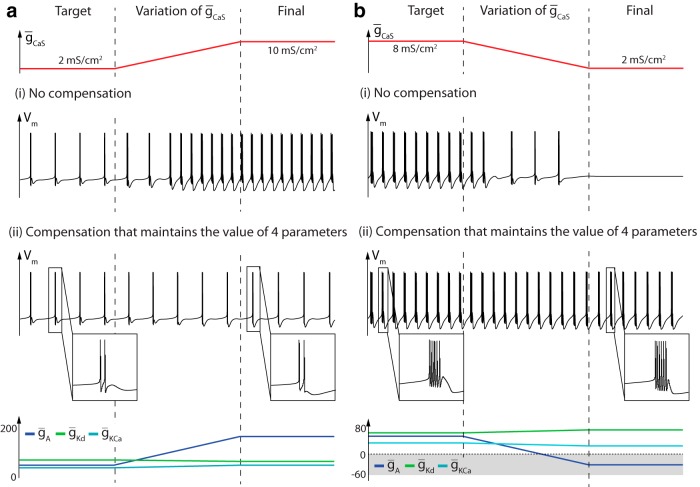
Compensation mechanism derived from the sensitivity analysis. ***a***, ***b***, Variation of the slow calcium channel density (top trace) and membrane potential variation over time in the absence and the presence of the compensation mechanism (center traces), and variations of channel densities involved in the compensation mechanism (bottom trace).

Figure 7 illustrates the neuronal spiking robustness that arises from this simple compensation mechanism. In Figure 7*a*, the density of slow calcium channels is increased fivefold (top red trace). Without any compensation mechanism, this variation strongly affects neuronal activity, which shows that the model is sensitive to this increase in calcium conductance (middle trace). In the presence of a compensation mechanism, the effect of the slow calcium channel variation is robustly silenced by changes in the maximal conductance of the potassium channels (bottom traces). The variations are consistent with the individual channel contributions illustrated in Figures 5 and 6. In particular, *ḡ_K,Ca_* increases to compensate for the positive contribution of *ḡ_Ca,S_* in the ultraslow timescale at spike threshold, whereas the increase of *ḡ_A_* compensates for the positive contribution of *ḡ_Ca,S_* as well as the negative contribution of *ḡ_K,Ca_* in the slow timescale at spike threshold. Finally, *ḡ_K,d_* decreases to correct the pathological negative effect of *ḡ_K,Ca_* in the slow timescale at up-state. Note that correlations in ion channel densities arising from our compensation mechanism are strictly linear (see Materials and Methods). This is a consequence of the linear dependence in conductance parameters of conductance-based models and agrees with many experimental and computational observations (Schulz et al., 2007; Zhao and Golowasch, 2012; O’Leary et al., 2013; O’Leary et al., 2014)

Figure 7*b* illustrates the effects of a fourfold decrease of the density of slow calcium channels. Here again, the model shows the sensitivity of the neuronal activity to this variation in the absence of compensation mechanisms (center trace). The initial neuronal activity, alternatively, is robustly maintained with the compensation mechanism (bottom traces). In this example, however, the A-type potassium conductance becomes negative (gray zone in the bottom of Fig. 7), which is a nonphysiological scenario. This illustration highlights that the dynamical activity is determined by the dynamic input conductances, regardless of how they are shaped by the individual ion channel conductances. It also points to a physiological limitation of the compensation mechanism. In this example, the slow positive feedback contribution of slow calcium channels is an essential component of the firing pattern and the neuron lacks an alternative current to generate the positive feedback that is necessary to compensate for the lack of slow calcium channels. Mathematically, the required positive feedback is eventually provided by changing the sign of the A-type potassium, thereby reversing the negative sign of the physiological feedback. This scenario indicates that a physiological compensation mechanism is limited by the availability of ion channels that can shape the dynamic input conductances similarly to the missing channel.

Another limitation of the proposed compensation mechanism is that it focuses on a few points in the dynamic input conductances. Significant differences in the dynamic input conductances away from those particular points can affect the performance of the compensation. More fundamentally, compensation for changes in one channel by changes in only one other channel is never perfect because each channel curve is localized around particular potentials. The robustness of the compensation mechanism is therefore conditioned by the overlap of the different sensitivity curves. This is particularly true in the slow timescale where the sensitivity of several channels is highly localized.

**Fig. 8:**
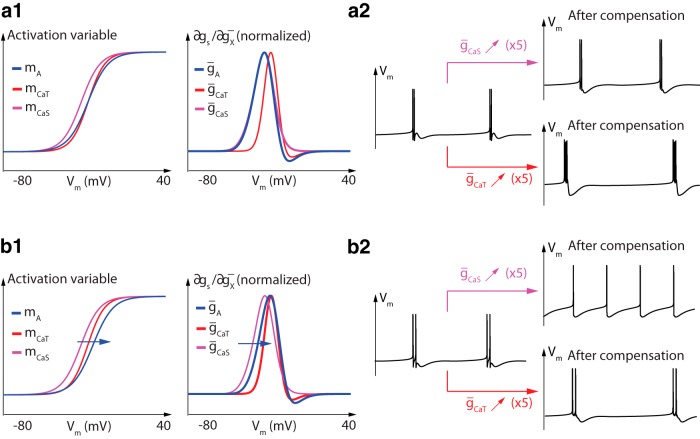
Colocalization of sensitivity curves, not activation curves, is crucial for robust compensation. ***a1***, Activation functions (left) and sensitivity functions (right) of A-type potassium channels (blue), T-type calcium channels (red), and slow calcium channels (magenta). ***a2***, Membrane potential variations over time before (left) and after a fivefold increase in slow calcium channel density (top right) and T-type calcium channel density (bottom right) in the presence of the compensation mechanism. ***b1***, ***b2*,** Same as ***a1*** and ***a2***, respectively, after a right shift of 4.5 mV in A-type potassium channel activation function.

Figure 8 illustrates the important role of the colocalization of the sensitivity functions in compensation mechanisms. Figure 8*a2*, top trace, illustrates that A-type potassium channels more easily compensate for changes in slow calcium channel density than in T-type calcium channel density, which has also been observed previously in a similar model (O’Leary et al., 2014). This is because the sensitivity ranges are much better colocalized in the first case. In fact, the sensitivity curves of slow calcium channels and A-type potassium channels almost overlap, allowing for almost exact mutual compensation (Fig. 8*a1*, right). It should be stressed that it is the colocalization of sensitivity functions, not of activation functions, that matters for the compensation. Figure 8*a1*, left, illustrates the better overlap of activation functions of A-type and T-type calcium channels compared to slow calcium channels, but this overlap is less relevant for compensation.

To further substantiate that it is the colocalization of sensitivity functions that matters for compensation mechanisms, we shifted the activation function of A-type potassium channels 4.5 mV towards more depolarized potentials (Fig. 8*b1*, left). As a result, the sensitivity curve of A-type potassium channels now roughly colocalizes with the one of T-type calcium channels, whereas the colocalization with slow calcium channels is lost (Fig. 8*b1*, right). The consequence of this adjustment on neuronal activity is that the compensation mechanism is now much more efficient against density variations in T-type calcium channels than against density variations in slow calcium channels (Fig. 8*b2*). Here again, the prediction cannot be made from the activation curves, which highlights the specific role of the sensitivity curves in the analysis of robustness and homeostatic mechanisms.

## Discussion

### Dynamic input conductances link ion channel distribution and neuronal activity

This paper introduces the concept of dynamic input conductances. These dynamic input conductances are shown to contain all the necessary information to link changes in ion channel density to their effect on neuronal spiking. Although these quantities are conceptual, they have the potential to provide new intuitions on how ion channels organize to modulate or maintain a target firing pattern.

We propose a method to either measure dynamic input conductances in a voltage-clamp experiment or extract them from an arbitrary conductance-based model. Dynamic input conductances provide a dynamic analog of the classical static input conductance, the basic tool to study the sensitivity of stable steady-states. Dynamical sensitivity analysis of arbitrary activity described by a set of nonlinear differential equations is computationally impractical. Dynamic input conductances circumvent this difficulty by taking advantage of the property that neuronal activity is a succession of well-defined temporal events such as spikes and bursts of spikes. The underlying timescale separation allows us to reduce the dynamic sensitivity analysis of the neuronal model to the sensitivity analysis of few quasi-static input conductances, one per timescale. Three timescales (and therefore a decomposition of the static input conductance into three distinct dynamic input conductances) were selected in the present paper to study separately the sensitivity of the fast upstroke of action potentials (fast timescale), the sensitivity of the slow downstroke of action potentials (slow timescale), and the sensitivity of the ultraslow adaptation of spiking (ultraslow timescale). A simple computational algorithm was proposed to distribute the contributions of a given ion channel in the three timescales and to compute the corresponding three dynamic input conductances from an arbitrary conductance-based model. Illustrations of the method on the STG model (Turrigiano et al., 1995; Liu et al., 1998; Goldman et al., 2001), a model that has served many previous studies of modulation and robustness of neuronal spiking, showed that the three dynamic input conductances are highly prototypical curves determined by a few parameters and that those few parameters shape important temporal properties of the neuronal activity.

### Dynamic input conductances as model reduction for sensitivity analysis

The parameterization of the dynamic input conductances by a few key parameters (such as their values near spike threshold and peak amplitudes) that control the dynamic activity can be thought of as an analog of reduced modeling of neuronal activity, but with the objective of sensitivity analysis rather than simulation. The conventional objective of neuronal reduced modeling is to extract a low-dimensional dynamical model (with few abstract state variables) that approximates the time activity of the corresponding high-dimensional quantitatively detailed conductance-based model with all the gating variables accurately modeled. In the present paper, the objective is not simulation but sensitivity analysis. As a consequence, the proposed method aims to extract a few meta-parameters not for simulation but to concentrate the parametric sensitivity analysis onto a few scalar quantities. The main advantage of the reduction process is the simplicity and robustness of the resulting analysis: the sensitivity analysis of the meta-parameters amounts to computing their derivatives (i.e., infinitesimal sensitivity) with respect to arbitrary parameters of the original model, an elementary mathematical operation that is robust because it is about qualitative shaping properties of the dynamic input conductances and does not suffer from the curse of dimensionality of high-dimensional models.

This is to be contrasted with more generic and non-local methods of sensitivity analysis such as extensive Monte-Carlo simulations of the high-dimensional quantitative model (Achard and De Schutter, 2006; Prinz, 2007; Prinz, 2010; Doloc-Mihu and Calabrese, 2014). Another key advantage of the reduction process is that the proposed meta-parameters provide a precise bridge between the quantitative biophysical parameters of the conductance-based model and qualitative properties that have a clear dynamical interpretation as local sources of positive or negative feedback in a given timescale. The importance of the physiological interpretation of the meta-parameters should not be underestimated. It facilitates the detection of aberrant results due to, for instance, modeling errors, and it readily allows for physiological predictions from the mathematical results, in contrast to the results of a general but somewhat blind high-dimensional sensitivity analysis.

### Sensitivity analysis of dynamic input conductances predicts robustness of neuronal spiking

For all its computational advantages, the inherent fundamental limitation of local sensitivity analysis (through derivatives, i.e., infinitesimal parameter variations) is that it might fail to predict the consequences of possibly large parameter variations encountered in practice. Although a classical and highly successful analysis tool in engineering, the success of local sensitivity analysis depends on the mathematical object under study, and its practical significance must be assessed empirically. The illustrations on the STG model here are encouraging in that regard. They suggest that local sensitivity analysis of dynamic input conductances has high predictive value regarding the distinct role of distinct channels in regulation and robustness of neuronal spiking, even for channel density variations exceeding those observed in experiments.

This success is perhaps not accidental in that the selected meta-parameters have a clear physiological interpretation and are supported by a rigorous mathematical analysis in previous work of the theoretical modulation and robustness capabilities of an arbitrary conductance-based model. For this reason, we believe that the methodology of the proposed method has general value beyond the specific illustrations chosen for the present paper. There is much room for further tailoring of the proposed computational algorithms to specific sensitivity analysis applications that could, for instance, include more than three timescales, different meta-parameters extracted from the dynamic input conductances, and network rather than single-cell neuronal activity. Also, the present paper focuses on variations of maximal conductances, i.e., channel density variations, as a primary source of modulation and robustness, but the sensitivity analysis can be applied to any parameter. At its core, it only rests on the fundamental assumption that the analyzed temporal activity can be decomposed as a succession of temporal events in distinct timescales.

### Sensitivity analysis provides mathematical insight on the richness and robustness of neuron excitability

The physiological relevance of our sensitivity analysis was assessed on a particular model of a specific organism that has served many earlier computational and experimental studies of neuronal modulation and robustness. We illustrated how the proposed sensitivity analysis provides insight on an apparent paradox between neuron sensitivity and robustness: a tiny variation in the conductance of a specific ionic channel, through the action of a specific neuromodulator, can, for instance, drastically affect the neuronal activity while large variability of the same parameter can be almost perfectly compensated for by covariation of other ion channel densities, provided that they have an overlapping sensitivity range in the affected timescales. Such predictions are relevant for experimental studies of neuromodulation and could assist the design or interpretation of novel experiments. The discussion contrasting the role of colocalization of activation ranges versus sensitivity ranges in compensation mechanisms is an example of prediction that is very much in line with recent experimental observations in mammalian dopamine neurons (Amendola et al., 2012): half-activation potentials of A-type potassium channels and HCN channels significantly vary from cell to cell but the covariation of the two channels is very stable across populations. Because the proposed analysis is computationally elementary and versatile, it can serve as a useful computational tool in resolving significant neurophysiological problems.

## Figures and Tables

**Figure R1:**
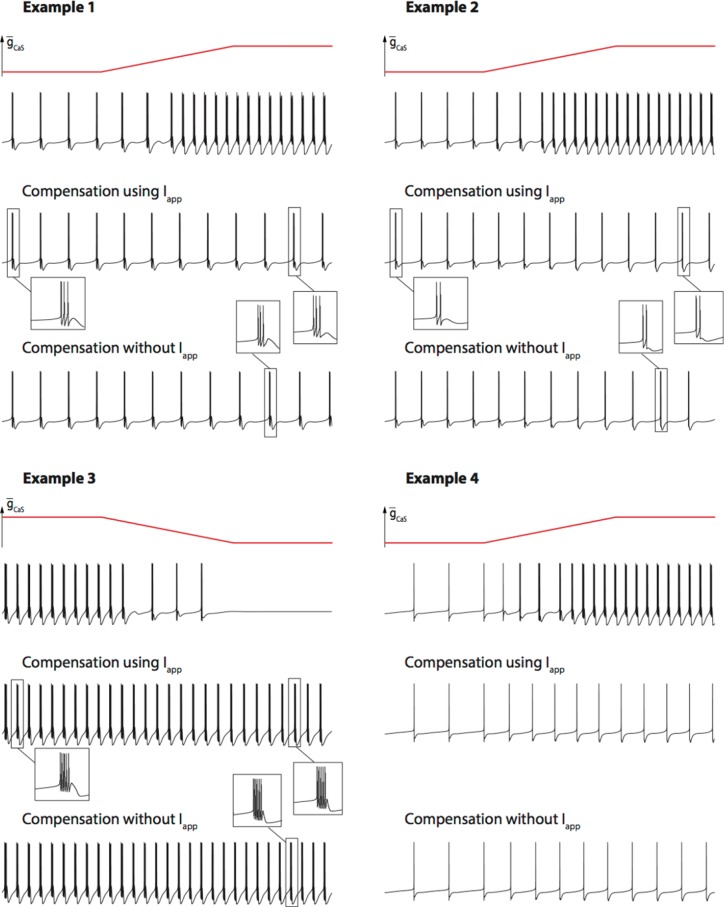
Comparison of the performances of the compensation mechanism against variations in gcas with and without variations of the applied current I_app_.

**Figure R2:**
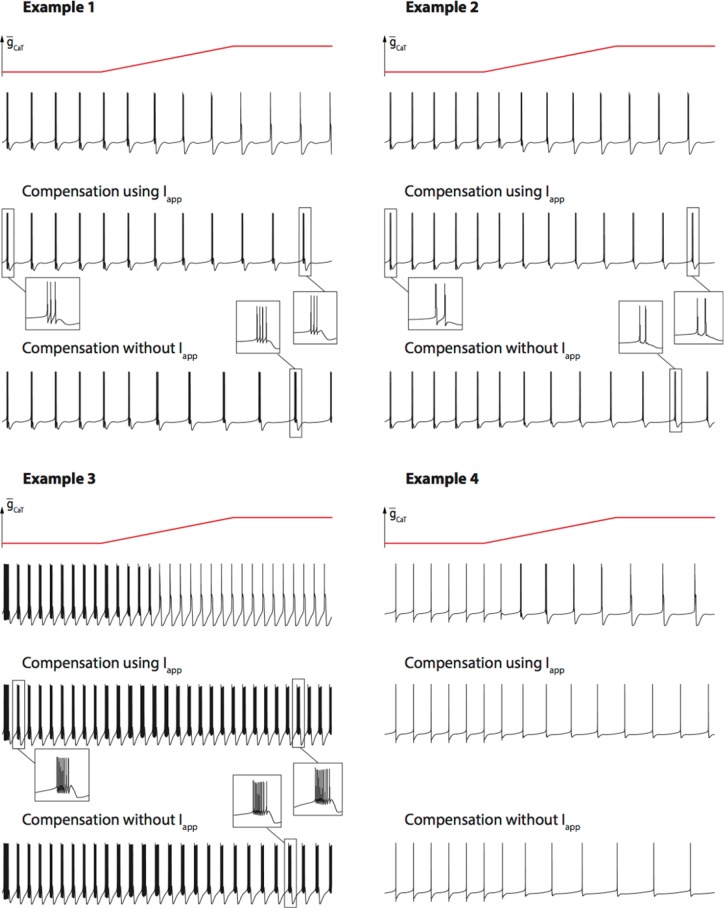
Comparison of the performances of the compensation mechanism against variations in gcat with and without variations of the applied current I_app_ in the robust case (half-activation potential of I_k_,A shifted as in Fig. 8 of the main manuscript).

**Figure R3:**
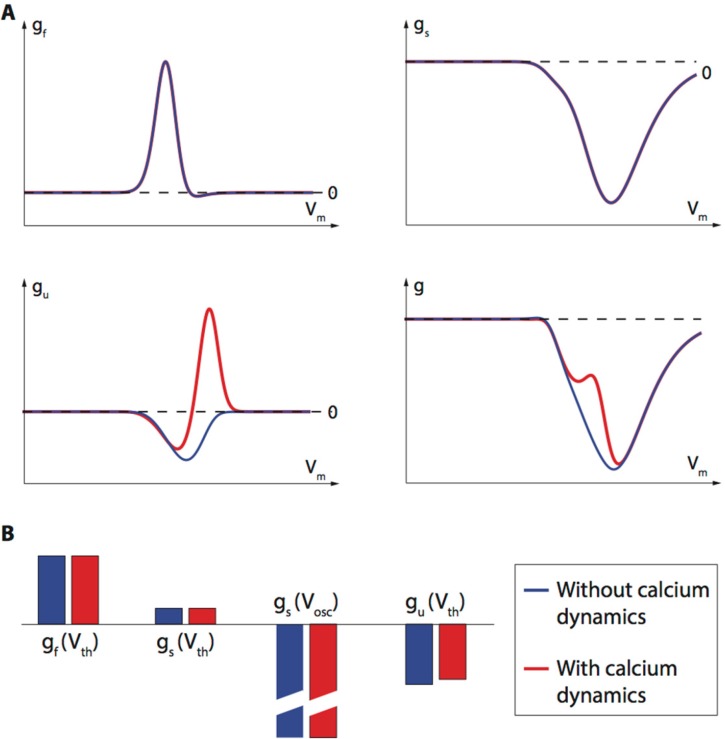
Comparison of the shape of the dynamic input conductances when calcium dynamics are neglected (blue) or included (red). (**A**) Dynamic conductances of one illustration case. (**B**) Values used in the compensation mechanism. The main effect of the calcium dynamics is to add a positive feedback in the ultraslow timescale at suprathreshold potential. Including calcium dynamics does not significantly affect the values used in the compensation mechanism in our illustration examples.

## Synthesis

The decision was a result of the Reviewing Editor Marlene Bartos and the peer reviewers coming together and discussing their recommendations until a consensus was reached. A fact-based synthesis statement explaining their decision and outlining what is needed to prepare a revision is listed below. The following reviewers agreed to reveal their identity: Ronald Calabrese and Scott Hooper

The manuscript was viewed by two reviewers and the editor. The reviewers and the editor judge the study as an important contribution to the understanding of how membrane conductances define neuron activity. However, as you can see from the reviewer comments there are three major concerns which should carefully addressed by the authors. A major concern was related to the understanding of figure 1 and the related text. A further major concern was related to the control burst frequency and finally the intracellular Ca2+ concentration. I would like to ask you to respond to all comments and questions, specifically to those related to figure 1.

### Review #1:

The authors present an analysis of dynamic input conductances in a well-established model of a bursting neuron from the crustacean STN. The idea of the dynamic input conductance is novel; it decomposes the active conductances of the model into n (3 in the current case) different time scales and extracts meta parameters in each time scale that are dependent on explicit model parameters (in this case maximal conductances of particular ion channels). The results of the decomposition lend themselves to sensitivity analysis and are predictive for modulation and homeostasis by compensation. The simulations are well done and the analysis is appropriate. The writing is clear and the figures well designed. The novelty of the work lies not in the specific predictions made for the model analyzed, but in the potential generalization of the method developed.

Concerns

1. Several aspects of the analysis seem trivial in that other methods, already having been employed, have led to similar predictions in the analyzed system and others. Since the paper is meant mainly as a methods paper this is not a major problem.
2. When speaking of modulation one should always specify what characteristic is being modulated. In biological systems this is important. Modulators do not function to change parameters but to change burst characteristic in a behaviorally relevant way. Please specify what burst characteristic one is seeking to modulate in your simulation experiments.
3. I found the lack of consideration of the leak current and its reversal potential to be a flaw in the analysis, especially since injected current was used in an unspecified way in the experiments of Fig 7 (also Fig 8???). Please specify why leak current was not considered and why its conductance (reversal potential) is not considered a relevant parameter for the dynamic input conductance. How was injected current varied in the experiments presented? What happens if it is not varied?
4. Several minor comments (no need to address these directly in any rebuttal but potentially useful in revision) and edits (mainly typo fixes and slight edits of the writing) are added to the manuscript pdf file.

### Review #2:

I have only three major concerns.

1)Fig. 1 and associated text: These are central to readers understanding the manuscript, and I, at least, did not find them clear. The text description is brief and technical, and the figure does not really help. For the "static" IV curve, when is the current being measured? For the dynamics, when is the current being measured? Given that they are presumably being measured at different times, why is it that the dynamic curves should add up to being the static curve? Or am I completely misunderstanding what is going on here? I am certain that what the authors did is correct, I just can't understand what it was that they did from the text and this figure. A small point, in the left panel, there should be two Vm traces, since the sub-threshold and suprathreshold Im's traces were induced by differently sized voltage traces. Explaining these curves is absolutely critical and, in my opinion, the manuscript should not proceed to publication until this task is better done.
2)The authors do not write much about control of burst frequency. This should, and does, be controlled by the ultraslow dynamic curve (Fig. 5 b2; Fig. 7a, b, top panels). Given the importance of burst frequency in motor pattern generation (fast vs. slow walking, etc.), the authors should spend some space writing about their results relevant to this issue.
3)I am concerned about the intracellular Ca concentration remaining constant in this work. The authors brush this off, but do so without compelling arguments as to why it should be correct to do so. IKCa depends on [Ca]i, and [Ca]i will change slowly, thus likely substantially altering the IKCa activity from that shown. My concern is heightened by the author's mention in the Discussion that "local sensitivity analysis is dependent on the mathematical studied object". It is thus possible that more accurate [Ca]i would substantially alter manuscript conclusions, in particular, might no longer permit the clean separation of conductance into the three time classes or otherwise interfere with the predictive power of this approach. I think it is therefore important, if the authors want this work to have the influence it deserves, that they either show that models that include [Ca]i are qualitatively similar to the present results, or give more convincing arguments that this should be the case.
  I made many minor writing corrections. I have attached an MS Word file with these, as that was easier than my typing them here. Beware of this file! PDF to Word translation is far from perfect and there are therefore many typos and other errors in the text. This file is just for the authors to find where I made changes; they should use it as a guide to make changes in their own file, not use my file as their beginning point. A few issues that they may find useful in future writing
  a) only animals behave or have behavior; neurons and models have activity
  b) i.e. and e.g. are always followed by commas ("that is", "for example" are always followed by commas).
  c) Please use active voice whenever possible. It is much shorter and easier to read.
  d) Unless it begins a sentence, "Figure" is always abbreviated to "Fig."
  e) Things always compensate for something, not just compensate: "The student compensated for his academic inadequacies by excelling at sports.", not "The student compensated his academic inadequacies by excelling at sports."

## Author Response

### Synthesis

The manuscript was viewed by two reviewers and the editor. The reviewers and the editor judge the study as an important contribution to the understanding of how membrane conductances define neuron activity. However, as you can see from the reviewer comments there are three major concerns which should carefully addressed by the authors. A major concern was related to the understanding of figure 1 and the related text. A further major concern was related to the control burst frequency and finally the intracellular Ca^2+^ concentration. I would like to ask you to respond to all comments and questions, specifically to those related to figure 1.

We would like to thank the editor and reviewers for their constructive and insightful comments, which helped us addressing important issues in the revised version of the manuscript. Answers to reviewer comments are given in red. Some figures that support these answers but were found not essential for the manuscript are attached at the end of this document. They are labelled « Fig. RX ».

Fig. 7b of the manuscript has also been slightly corrected (a small confusion has been found in the code). The compensation is actually better than previously shown.

### Review #1

The authors present an analysis of dynamic input conductances in a well-established model of a bursting neuron from the crustacean STN. The idea of the dynamic input conductance is novel; it decomposes the active conductances of the model into n (3 in the current case) different time scales and extracts meta parameters in each time scale that are dependent on explicit model parameters (in this case maximal conductances of particular ion channels). The results of the decomposition lend themselves to sensitivity analysis and are predictive for modulation and homeostasis by compensation. The simulations are well done and the analysis is appropriate. The writing is clear and the figures well designed. The novelty of the work lies not in the specific predictions made for the model analyzed, but in the potential generalization of the method developed.

#### Concerns

1. Several aspects of the analysis seem trivial in that other methods, already having been employed, have led to similar predictions in the analyzed system and others. Since the paper is meant mainly as a methods paper this is not a major problem.
  The contribution of the paper is indeed mainly in a new methodology. We purposely illustrate its prediction capacities in a well studied neuron model for comparison purposes, and most of the illustrative results are in agreement with what have been done in the literature, with the exception of results presented in the last section (and especially the last figure), which we think are original.
2. When speaking of modulation one should always specify what characteristic is being modulated. In biological systems this is important. Modulators do not function to change parameters but to change burst characteristic in a behaviorally relevant way. Please specify what burst characteristic one is seeking to modulate in your simulation experiments.
  Except for one place where “modulation” was misused and has been replaced by a different terminology (see Fig. 7), the use of the terminology “modulation” throughout the paper is always in the sentence “modulation and robustness”. The paper is not about a specific modulation function, but rather about a methodology that quantifies how variations in the maximal conductances can simultaneously achieve robustness and modulation. The only figure that explicitely suggests how changes in the dynamic conductances participate in the modulation is Figure 4. The revised figure illustrates how to modulate the burst frequency (and this revision was motivated by Q2 from Rev 2).
3. I found the lack of consideration of the leak current and its reversal potential to be a flaw in the analysis, especially since injected current was used in an unspecified way in the experiments of Fig 7 (also Fig 8???). Please specify why leak current was not considered and why its conductance (reversal potential) is not considered a relevant parameter for the dynamic input conductance. How was injected current varied in the experiments presented? What happens if it is not varied?
  The reviewer is right that the original version of the manuscript is silent about the role of the leak current whereas it is well known that varying the leak current may affect the excitability of the neuron. The reason for this apparent contradiction is that, similarly to the applied current, the leak current affects the (static) I/V curve but that it does not affect the input dynamic conductances defined in this paper. The role of the I/V curve on excitability has been well studied in the literature and is not repeated in our paper which, instead, insists on the importance of dynamic properties in the analysis of excitability. The leak current is part of the capacitive currents that can be considered as instantaneous compared to the fast, slow, and ultraslow ionic currents analyzed in our paper. It is customary to compensate for the capacitive current in a voltage clamp experiment and we chose to do so in our manuscript in order to concentrate our analysis on the ionic currents. A new sentence has been added at the end of the section on dynamic conductances to clarify that omission.
  The compensation mechanisms studied in Figure 7 and 8 indeed compensate both for the static I/V curve (through the applied current) and the dynamic conductances. It should be noted that the importance of maintaining the static current unchanged (through an adjusted Iapp) is modest as compared to the other parameters in our illustration examples. This is validated by Fig. R1 and Fig. R2 below, where we performed the same simulations without adjusting the applied current. It should also be noted that many currents, not included in our study, could account for the physiological compensation of the variation of the static current (i.e. persistent sodium and H-type currents, for instance). A new sentence has been added in that section in order to clarify this point.
4. Several minor comments (no need to address these directly in any rebuttal but potentially useful in revision) and edits (mainly typo fixes and slight edits of the writing) are added to the manuscript pdf file.
  Corresponding corrections have been made in the manuscript.

### Review #2

I have only three major concerns.

1. Fig. 1 and associated text: These are central to readers understanding the manuscript, and I, at least, did not find them clear. The text description is brief and technical, and the figure does not really help. For the "static" IV curve, when is the current being measured? For the dynamics, when is the current being measured? Given that they are presumably being measured at different times, why is it that the dynamic curves should add up to being the static curve? Or am I completely misunderstanding what is going on here? I am certain that what the authors did is correct, I just can't understand what it was that they did from the text and this figure. A small point, in the left panel, there should be two Vm traces, since the sub-threshold and suprathreshold Im's traces were induced by differently sized voltage traces. Explaining these curves is absolutely critical and, in my opinion, the manuscript should not proceed to publication until this task is better done.
  We acknowledge that the explanation of how dynamic conductances can be determined is crucial to the message of the paper. For this reason, in addition to the model-based methodology explained in the original version, the revised version includes an ‘experimental voltage-clamp protocol’ to determine the dynamic conductances. Our hope is that this voltage-clamp ‘experiment’ and the new figure provide a clear account of how the static conductance can be decomposed as a superposition of dynamic conductances in different time scales. Answer to this concern also tackles concern 3 (inclusion of calcium dynamics in the dynamic input conductances). It can be observed that the dynamic input conductances obtain through the (simulated) voltage-clamp experiment are consistent with the curves analytically extracted from the conductance-based model (shown in pannel (c) of the new Fig. 1, as well as Fig. R3 below). We hope that the new Figure and revised text will answer the legitimate questions of the reviewer.
2. The authors do not write much about control of burst frequency. This should, and does, be controlled by the ultraslow dynamic curve (Fig. 5 b2; Fig. 7a, b, top panels). Given the importance of burst frequency in motor pattern generation (fast vs. slow walking, etc.), the authors should spend some space writing about their results relevant to this issue.
  We were indeed not exhaustive in describing the roles of the different dICs in neuromodulation, and in particular regarding the role the ultraslow curve. We answer this concern by adding an illustration of the relationship between the ultraslow dIC and burst frequency in Fig. 4 of the manuscript.
3. I am concerned about the intracellular Ca concentration remaining constant in this work. The authors brush this off, but do so without compelling arguments as to why it should be correct to do so. IKCa depends on [Ca]i, and [Ca]i will change slowly, thus likely substantially altering the IKCa activity from that shown. My concern is heightened by the author's mention in the Discussion that "local sensitivity analysis is dependent on the mathematical studied object". It is thus possible that more accurate [Ca]i would substantially alter manuscript conclusions, in particular, might no longer permit the clean separation of conductance into the three time classes or otherwise interfere with the predictive power of this approach. I think it is therefore important, if the authors want this work to have the influence it deserves, that they either show that models that include [Ca]i are qualitatively similar to the present results, or give more convincing arguments that this should be the case.
  The revised version of the paper includes the calcium dynamics and the intracellular calcium concentration is now treated as an extra state variable of the conductance-based model. remains a variable in all simulations. The reviewer is right in that calcium dynamics can influence the ultralslow dynamic conductance significantly: if calcium channel densities are large enough, the ultraslow dynamic conductance exhibits a region of large positive feedback (see Fig. R3A below). This positive feedback comes from the interaction between calcium channel inactivations and the dynamics of intracellular calcium concentration in the ultraslow timescale : starting from a suprathreshold potential, a depolarization of the membrane induces the inactivation of the calcium channels, which in turn reduces the amplitude of the calcium-activated potassium current through a reduction in intracellular calcium concentration, and therefore further potentiates the initial depolarization.
  Because it occurs in the suprathreshold region, this significant effect on the ultraslow dynamic input conductance barely affects the conclusions of our study. This is because for the ultraslow timescale (and any slower timescale), the value of the dynamic input conductance only matters in the suthreshold region (excursions of the membrane potential in the supratreshold region are too fast to recruit the ultraslow dynamics). Finally, adding calcium dynamics in the computation of the dynamic input conductances barely affects the parameter values used in the compensation mechanisms of Fig. 7 and Fig. 8 (see Fig. R3B below).
  I made many minor writing corrections. I have attached an MS Word file with these, as that was easier than my typing them here. Beware of this file! PDF to Word translation is far from perfect and there are therefore many typos and other errors in the text. This file is just for the authors to find where I made changes; they should use it as a guide to make changes in their own file, not use my file as their beginning point. A few issues that they may find useful in future writing
  a) only animals behave or have behavior; neurons and models have activity
  b) i.e. and e.g. are always followed by commas ("that is", "for example" are always followed by commas).
  c) Please use active voice whenever possible. It is much shorter and easier to read.
  d) Unless it begins a sentence, "Figure" is always abbreviated to "Fig."
  e) Things always compensate for something, not just compensate: "The student compensated for his academic inadequacies by excelling at sports.", not "The student compensated his academic inadequacies by excelling at sports."
    Corresponding corrections have been made in the manuscript.
